# Questions on causality and responsibility arising from an outbreak of Pseudomonas aeruginosa infections in Norway

**DOI:** 10.1186/1742-7622-5-22

**Published:** 2008-10-23

**Authors:** Bjørn G Iversen, Bjørn Hofmann, Preben Aavitsland

**Affiliations:** 1 Norwegian Institute of Public Health, Oslo, Norway; 2 University College of Gjøvik, Faculty of Health, Care and Nursing, Gjøvik, Norway; 3 University of Oslo, Department of General Practice and Community Medicine, Section for Medical Ethics, Oslo, Norway

## Abstract

In 2002, Norway experienced a large outbreak of *Pseudomonas aeruginosa *infections in hospitals with 231 confirmed cases. This fuelled intense public and professional debates on what were the causes and who were responsible. In epidemiology, other sciences, in philosophy and in law there is a long tradition of discussing the concept of causality. We use this outbreak as a case; apply various theories of causality from different disciplines to discuss the roles and responsibilities of some of the parties involved. Mackie's concept of INUS conditions, Hill's nine viewpoints to study association for claiming causation, deterministic and probabilistic ways of reasoning, all shed light on the issues of causality in this outbreak. Moreover, applying legal theories of causation (counterfactual reasoning and the "but-for" test and the NESS test) proved especially useful, but the case also illustrated the weaknesses of the various theories of causation.

We conclude that many factors contributed to causing the outbreak, but that contamination of a medical device in the production facility was the major necessary condition. The reuse of the medical device in hospitals contributed primarily to the size of the outbreak. The unintended error by its producer – and to a minor extent by the hospital practice – was mainly due to non-application of relevant knowledge and skills, and appears to constitute professional negligence. Due to criminal procedure laws and other factors outside the discourse of causality, no one was criminally charged for the outbreak which caused much suffering and shortening the life of at least 34 people.

## Introduction

In 2002, we traced the source of a large outbreak of *Pseudomonas aeruginosa *infections to contaminated mouth swabs extensively used in Norwegian health care [1]. The investigation revealed many weaknesses and errors in the chain from production to use [2].

During and after the outbreak investigation, questions of causality, responsibility and liability were raised: Who and what caused the outbreak, who were responsible for the extent of the outbreak, could the damages have been mitigated by acting sooner or differently, should anyone be punished? Questions of causality, responsibility and blame have always been a part of the history of infections. Two examples are the debate on where the Spanish flu came from and who was responsible for starting the Aids epidemic.

The concept of causality is intuitively simple and yet so intricately complex. In epidemiology causality has been hotly debated [3-11]. In philosophy of science there is a long tradition of discussing both the content of the term and how to achieve knowledge about the association of events [12]. In law, decisions on responsibility and liability rests on whether a specific action has *caused *specific harm or loss to another, and jurisprudence frequently defers to science in order to settle issues of causality [13-16]. However, not only is the discourse of causality in the philosophy of science interesting for law, reciprocally the debate on legal causation, especially in tort law, is useful for the scientists and the philosophers of science. In all these three disciplines (science, philosophy and law) and in practical life this discourse has implications for placing moral responsibility, blame, honour and dishonour. Consequently the general debate on causality is of interest both for scientists, manufacturers, and lawyers, as for the general public because it influences moral as well as professional norms.

In this article, we will use the outbreak of *P. aeruginosa *infections to illustrate the relevance of various theories of causality and discuss the role of the different participants. Then we will discuss the responsibility and fallibility for two of the main actors in the outbreak.

### Setting the scene

**Figure 1:**
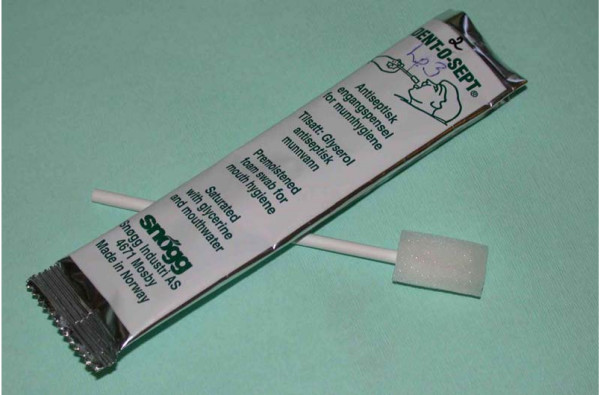
The Dent-O-Sept mouth swab.

Late February 2002, the Norwegian Institute of Public Health (NIPH) was alerted of a possible increase in the number of *Pseudomonas *infections in clinical wards of Norwegian hospitals [1]. After a strenuous outbreak investigation, on 8 April 2002, the outbreak strain was isolated from a domestically produced mouth swab for use in health care, called "Dent-O-Sept" (figure 1). The finding was publicised, the product was recalled, and the production ceased permanently.

The outbreak strain was detected in swabs from 12 batches produced in 2001 and 2002 [2] and from the production line in the factory. An audit of the producer revealed several breaches of production regulations [17]. Health care institutions reported some extent of non-proper reuse of the swabs and weaknesses in their purchasing systems.

The strain was detected in 231 patients from 24 hospitals, of whom 71 (31%) died while hospitalised; all had severe underlying disease. For at least 34 patients the investigators concluded that the *P. aeruginosa *infection probably contributed to the patient's death [1]. No one was found criminally liable for the outbreak.

Two of the authors (BGI and PA) were responsible for the outbreak investigation at the Norwegian Institute of Public Health [1,2]. After six years have passed we feel that we can give a balanced review of the causes of the outbreak but will abstain from evaluating our role in it.

So, what was the cause of the outbreak, and who were responsible? Let us first examine the issues of causation from a scientific point of view, and then relate them to the legal issues of responsibility and liability.

## Analysis

Before presenting theories on causality, responsibility and liability we need to define what was caused, i.e. what was the epidemiological outcome. We have asked "what caused the outbreak", but "the outbreak" is a rather diffuse concept and consists of the sum of individuals who each had their own set of factors contributing to them being included. Although the attention was brought to the individual cases by clinical manifestations (infections), we included in the outbreak all patients with genotypically identical strains of *P. aeruginosa*, irrespective of survival or severity of disease [1]. For this analysis we will make it clear which of the four different outcomes we have in mind; 1. being a case as defined in the outbreak investigation, 2. having a *P. aeruginosa *infection, irrespective of type of strain, 3. dying from *P. aeruginosa *infection or 4. the outbreak as a whole.

### Causality

We often say that one thing causes another, like "rain causes flooding" and "smoking causes cancer", although it is not always true. We don't have a flood every time it is raining, and flooding can have other reasons than rain: a broken water pipe for example.

The philosophical basis of the dominant approach for testing theories in medicine is the hypothetico-deductive model as described by for example David Hume and Karl Popper. According to this model it is impossible to achieve absolute proof for a scientific hypothesis; tests performed can only corroborate or falsify the hypothesis. Consequently one can never prove causality between factors and an outcome, only strengthen or weaken a proposed association. In this tradition Sir Austin Bradford Hill listed nine viewpoints from which to study the association of two variables in order to claim causation [3].

#### Causation in epidemiology

Classic epidemiology has been mainly backward looking, seeking an explanation to an event. In much of the 19^th ^century there was a profound debate on what caused many of the major diseases of the time, being it miasmata (stench or bad air) or contagions [18]. For a disease like cholera John Snow, the father of epidemiology, was in favour of the theory of a contagion which he called "morbid matter" [19]. Late in the 19^th ^century, a prominent microbiologist, Robert Koch, formulated a set of postulates that needed to be fulfilled in order to claim that a micro-organism caused a specific disease [20,21]. According to his postulates we need both necessary and the sufficient conditions to claim causal relationship between a microbe and a disease.

A century later MacMahon stated that there are two ways of classifying ill persons, either by *manifestational criteria *(grouping ill persons according to symptoms or clinical signs, e.g. common cold, schizophrenia or meningitis) or by *causal criteria *(grouping ill persons with respect to a specified experience believed to be a cause of their illness, e.g. lead poisoning or meningococcal disease) [22]. To have a *Pseudomonas aeruginosa *infection implies by name and definition causality of the bacterium.

#### Causation in law

The Norwegian legal system belongs to the French-German legal tradition which differs from the Anglo-American law in placing relatively more emphasis on statutory law than the judiciary legal institutions in the making of the legal framework. However, regarding tort law and causality the principles of the two legal systems are very similar. Likewise, both legal systems have a lower threshold for civil liability than criminal liability. There are several examples from recent history in Norway where the accused was found not guilty in the criminal court case but was convicted to pay economic compensation in a following civil lawsuit.

Causal connection in law is usually divided into two parts, "cause-in-fact" and "proximate cause" [16,23]. "Cause-in-fact" comes closest to what is regarded as causality in science. However, while science mostly deals with causal generalisation, law focuses more on causes of specific events. One standard method of establishing factual causation is the "but-for" test, aiming at excluding those factors that had no impact on the course of events. Another influential test for causation is the NESS-test, i.e. Necessary Element of a Sufficient Set test [23,24]. "Proximate cause", also called "adequate cause", embodies reasons for limiting the extent of legal responsibility and liability.

Additionally, deciding on legal responsibility and liability involves a counterfactual proposition, i.e., if a condition that in fact occurred had not occurred, then the outcome would have been different. Both the "but-for" test and the NESS test can be part of such counterfactual propositions. The "but-for" test asks: Would the consequences have occurred in these circumstances had the condition not been present? The NESS test asks: In these circumstances, is the condition a necessary member of a set of conditions that are together sufficient to produce the consequence [24]. Over-determination and joint determination are weaknesses of the "but-for" test, whereas lack of determination challenges the NESS test [22].

#### Counterfactual theories of causation in sciences

The central question in counterfactual theories of causation is "What would have happened if not event c had happened?" And the answer is: "If not event c had occurred, then the event e would not have occurred" [25]. Counterfactual reasoning can be used both in deterministic and probabilistic models. In daily life and in medicine counterfactual reasoning is extensively used. "If the needle hadn't been contaminated, the patient would not have acquired hepatitis." "If you hadn't been exposed to asbestos, you would not have contracted mesothelioma." Many of the epidemiological study designs have counterfactual thinking embodied [5]. In cohort studies we compare exposed and unexposed individuals for a certain risk factor. The unexposed group can be viewed as "what if this exposure did not occur". When calculating the attributable risk fraction, also called the etiological fraction, we assume that all association between the exposure studied and the outcome is causal, and in addition imply that if not the exposed group had been exposed, the rate of outcome among them would have been at the same level as among the unexposed.

#### Necessary, sufficient and complex conditions (determinism)

Many conditions are **necessary **for an event to occur. Every time the event occurs, the condition is present. A necessary condition for septicaemia is that one has blood; however, we do not say that having blood is the cause of sepsis. Owning the axe, which a person steals to kill a man, does not make you a murderer, even though the axe was a necessary condition for the man's death. This leads to the claim that causation is not given by the necessary conditions, although they are important, because if we can eliminate the necessary conditions, we can eliminate the problem.

Some conditions are **sufficient **to result in another: every time they occur, something else happens. Drinking a cup of hydrogen cyanide is a sufficient condition for death. However, other conditions may also result in the effect; not all deaths result from drinking hydrogen cyanide.

In complex situations many factors contribute to an effect, and there are logistic problems in that an event occurs some but not all times a constellation of factors occurs. To overcome this, Mackie introduced the so-called INUS condition of causation. An INUS condition for some effect is an Insufficient but Non-redundant part of an Unnecessary but Sufficient condition [26]. The NESS test described above is a clarification or specific instance of an INUS condition [23].

#### Determinism and probabilism

Causal determinism is based on the idea that that every event is necessitated by antecedent events and conditions together with the laws of nature [27]. According to causal determinism the causal relationships are invariant: Every time a certain configuration of conditions occurs, the outcome will be the same. We may have causal determinism even if the situation is complex and the outcome is hard to predict.

Probabilistic causality on the other hand claims that the causal relationship is probabilistic, and not invariant. That is, the outcome (effect) may vary according to probability distribution. Probabilistic theories of causation state that causes raise the probabilities of their effects [9].

In epidemiology, probabilistic approaches are most often used in the conceptual thinking of a relationship and in the statistical testing of the strength of association [9]. Here, Hill's set of nine viewpoints to explain the association between two variables are commonly used [3]. Only the one of temporal sequence of association is essential. This list of "Guidelines for causation" is more in tune with modern epidemiological science as they emphasize the strength of association rather than pure mechanical determinism. However, many have criticised Hill's list and in recent years there has been a resurge in the debate about causality [6-8,10,28]. Moreover, probabilistic graphical methods, such as Bayesian networks, may also be used in order to represent the probabilistic independencies between variables.

### Causation in the Dent-O-Sept case

We have now presented theories for causation, which can now be applied in a specific case, the Dent-O-Sept outbreak in Norway in 2002 [1,2].

#### Necessary and sufficient conditions

The *P. aeruginosa *bacterium is not a necessary and sufficient condition for the death of people. Neither is its presence in the production plant a necessary and sufficient condition for the presence of the bacteria in the product. Hence, if necessary and sufficient conditions are required for liability and moral responsibility, no one is responsible for the outbreak. Was the *P. aeruginosa *bacterium a necessary condition?

For patients involved in the Dent-O-Sept outbreak, having the outbreak strain of *P. aeruginosa *was **necessary **to be included as a case. If it was not for the *P. aeruginosa*, then there would have been no outbreak (i.e. "but-for"). But this is more a definition criterion for being a case and does not explain *why *the patient harboured this strain.

No single factor was absolutely required to be colonised or infected with the outbreak strain. The use of Dent-O-Sept was not a necessary condition for infection. Approximately one third of the cases had not used the swab directly. The outbreak investigators concluded that they probably were secondarily infected from contaminated environment or health care workers after the contaminated swabs had introduced the strain in the hospital environment. By including this indirect pathway, it is reasonable to claim that the contaminated swabs were a necessary condition for the patients to become infected. This is equivalent to outbreaks of gastroenteritis (e.g. salmonellosis) where the primary cases may be infected by contaminated food, but cases continue to occur by person to person transmission via the fecal-oral route even after the implicated food item has been removed. In these situations, we would usually say that the food contamination caused the whole outbreak, and not only the first cases.

*P. aeruginosa *is harmless to most people and in most instances. The large majority of patients with the outbreak strain of *P. aeruginosa *and all who died from the infection had severe underlying illnesses. To have a severe underlying illness was in practice a necessary condition to die from the outbreak strain. So, both the presence of *P. aeruginosa *in Dent-O-Sept and having an underlying illness were necessary conditions for dying from the infection. But there were other necessary conditions as well, such as being hospitalised, but this we would hardly call a cause of death. This illustrates the problem with necessary conditions: there are extremely many of them.

In the Dent-O-Sept outbreak no single condition was **sufficient **to result in infection with the outbreak strain. Given the large number of Dent-O-Sept swabs used in the period and the massive contamination, we believe that several thousand patients were exposed to the outbreak strain of *P. aeruginosa*. Only a few of them became lastingly colonised or infected. Hence, the contaminated Dent-O-Sept swab was not a sufficient condition for the outbreak of the infection.

**Figure 2:**
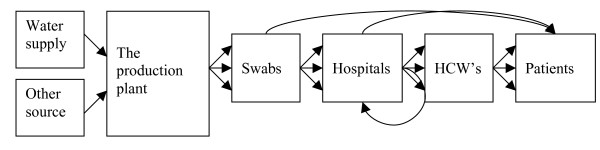
The direction of flow of the *Pseudomonas aeruginosa *bacteria from the production to the patients.

We may visualise a chain of unfortunate events necessary for the outbreak to occur: The outbreak investigation discerned the direction of flow of the *P. aeruginosa *bacteria from the production to the patients (figure 2). Can the links of this chain be seen as a series of necessary conditions that together are sufficient for the outbreak? However, the (necessary) conditions, such as the presence of *P. aeruginosa *in the mouth swabs and the patients' susceptibility, are not sufficient for the outbreak. There will not be an outbreak every time these conditions occur. Applying the concept of the INUS condition is helpful [26]. Using contaminated swabs was in itself insufficient but non-redundant, but together with other factors like the susceptibility of the patients, the infectious dose, and underlying illnesses, became sufficient to infect or colonise the patient.

According to this approach to causality we can find the altogether necessary and sufficient conditions for an event. If we know the conditions (making up the cause), the effect will occur. However, the challenge is that we do not know all the conditions and their complex interplay. For instance, we do not know the importance of the water quality during production or the significance of the reuse of the Dent-O-Sept for the outbreak.

#### Hill's postulates

In the Dent-O-Sept case, most of the points on Hill's list are helpful in order to claim association, at least to a certain degree (table 1). In addition to the traditional epidemiological measures of strength of association modern microbiology has developed tools to demonstrate associations. Various techniques of producing "fingerprints" of bacterial DNA have made it possible to identify identical strains of bacteria. In outbreak investigations these techniques have become very important to connect events and claim causality, and are similar to detection of human DNA in criminal investigations. When genotypically identical bacterial isolates were detected in the production plant, in some of the swabs and in the affected patients, we concluded that contamination of the production line caused the outbreak.

**Table 1:** Application of Hill's viewpoints on the causal association between the Dent-O-Sept swab and becoming colonised or infected during the outbreak

Hill's viewpoints	Application on the Dent-O-Sept outbreak
1. Strength of association	Strong.
	Association for having used the swab during hospitalisation and having the outbreak strain of *P. aeruginosa*, adjusted odds ratio 5.3.
	Detecting genotypically identical strains of the bacterium in patients, the product and in the production facility [1]
2. Consistency of association	Yes, to a large extent.
	However, other co-factors also needed to be in place, e.g. contamination of the particular swab and a susceptible patient. Due to secondary spread in the hospitals also patients who did not use the swab were infected.
3. Specificity of association	Yes, mostly.
	Use of contaminated swabs led to colonisation and some times to infection. Necessary co-factors were as above (2). The clinical manifestations of the *P. aeruginosa *infection varied widely.
4. Temporal sequence of association	Yes.
	However, the outbreak strain of the bacteria was found in six patients before the production of the first contaminated batch of swabs was detected [2].
	When the swabs were withdrawn from the marked the number of cases gradually diminished and disappeared.
5. Biological gradient	This was not tested but assumed. Reuse of the swabs may have increased the bacterial load and hence the risk of becoming infected.
6. Plausibility of association	Yes.
	The chain from contamination during production to infection is well described.
7. Coherence of association	Yes.
	There is no other hypothesis of explanations for the outbreak.
8. Experiment (reversibility)	Yes, a natural experiment.
	When the source was removed the number of cases gradually diminished to zero.
9. Analogy	Yes.
	There are many other outbreaks caused by medical devices. (References in [1])

#### Key actors

We will now in some more detail analyse key actors (table 2) and events in the light of causation and prepare for the next section on moral responsibility.

**Table 2:** The main participants in the Dent-O-Sept outbreak and some of their roles, responsibilities and actions.

**Participant**	**Role and responsibilities**
The producer "Snøgg AS"	• Produced the Dent-O-Sept swab
	• Did not adhere to the laws and regulations for production of medical devices
	• Lacked a quality assurance system for the production
	• Did not implement advise after external evaluation
	• Stopped the swab production as soon as the connection with the outbreak was established

The water supplier "Kristiansand municipality"	• Supplied drinking water to the producer
	• The *P. aeruginosa *bacterium may have been introduced into the production plant with the water

The hospitals	• Treated patients and procured medical devices
	• Many lacked quality assured systems for procurement, storage and use of medical devices
	• Many lacked systems for training of health care workers
	• Many had inadequate reporting systems for faulty medical devices

The health care workers	• Treated and cared for patients
	• Many reused the "single use" swabs contrary to the text on the wrap
	• Many did not report faulty medical devices

The patients	• Received medical treatment and care
	• Many were seriously ill and susceptible for contracting infections with the *P. aeruginosa *bacterium

The surveyor and investigator "Norwegian Institute of Public Health"	• Responsible for surveillance of infectious diseases and for outbreak investigations
	• There is no national surveillance system for *P. aeruginosa *infections

National administrative body "The Norwegian Directorate for Health and Social Affairs"	• Responsible for national administration within certain areas of the health care system
	• Responsible for the audit of the producer
	• Ignored the deadline to appeal the police's decision not to press charges.

**The producer, Snøgg AS **had a routine for cleaning and disinfecting the production line but had no quality assurance (QA) systems in place to control the production and verifying that the product was safe. Having QA systems is one of several requirements by Norwegian and EU law [29] to be able to mark the product "CE" (Communauté Européenne) which signifies that it complies with relevant EU-regulations and indicates it being safe. Some years prior to the outbreak, customers had periodically complained about discoloured swabs. In 1999, the producer commissioned an external evaluation and implemented some, but not all the advice given; among the latter was the advice to establish microbiological quality control of the final product [17].

An audit of the producer revealed serious breaches of production regulations [17]. Under strict liability, a party breaking the law may be legally responsible irrespective of whether any harm has been caused [24]. For instance, driving under the influence of alcohol is in Norway as in many other countries punishable by law even when no one is harmed.

The presence of the outbreak strain in the production facility was necessary for contamination to occur, but not sufficient as not all swabs were contaminated. This differed even in swabs produced at the same time of day on the same date. The main hypothesis of the outbreak investigation team was that bacteria-containing biofilm was randomly shed from the production equipment into the swab wraps [2]. Using the judicial, counterfactual "but-for" test: "But-for the absence of a microbiological quality control of the production equipment and the final product, would the contamination have been detected earlier and the outbreak avoided?" There is good reason to believe so. A total of 76 of 1565 swabs examined during the outbreak investigation contained the outbreak strain of *P. aeruginosa*, and more than 250 swabs (16%) contained one or more microbes (including the outbreak strain), also in swabs produced years before the Dent-O-Sept outbreak started [2]. Consequently, not abiding by the requirement to have QA systems including an effective microbiological control system can be seen as a cause in the legal sense for this outbreak to occur. The same goes if we apply a counterfactual NESS test.

The *P. aeruginosa *bacterium was also detected in a rubber hose leading from a water tap supplied with municipal water to a large steel tank used in the production [2]. In a press release the producer claimed that the municipal water company caused the outbreak [30]. This claim of the origin of the first bacterium could in retrospect not be verified. However, there are no requirements for tap water to be *Pseudomonas *or bacteria free. On the contrary, it is common microbiological knowledge that *P. aeruginosa *at times can be detected in water and soil [31,32]. The bacteria could have originated from other sources and contaminated the rubber hose. Using the "but-for" test on the water supply fails to show it to be a cause-in-fact due to the uncertainty of the origin of the first bacteria. Likewise, due to the uncertainty of the origin it fails a counterfactual NESS test. Hence, bacteria in the tap water cannot be seen as a legal cause of the outbreak.

Many of **the hospitals **had several deficiencies in their QA systems, for instance concerning the selection of which products to purchase; the actual procurement of the product; the logistic system for reception, distribution, storage, and use of the product [33]. Many of these deficiencies are in breach of national guidelines and legal regulations but did probably not have any influence on this particular outbreak. The bacterial load inside the wrap probably diminished over time as the bacteria cannot survive without oxygen. Consequently, the deficient logistics systems in the hospitals appear not to be a cause-in-fact of this outbreak. However, competent procurers in hospitals might have detected inferior products or the lack of documentation, such as the declaration of conformity which is required for all CE marked medical devices with the EU directive [29].

**Health care workers **(HCWs) are constantly told to be economical and prudent in the use of medical equipment. In many hospitals it was customary to reuse the swab in the same patient although it was clearly marked as single use equipment. In between cleaning the patient's mouth the swab was sometimes stored in a glass of water on the patient's night stand. This practice allowed rapid multiplication of bacteria on the swab. This unprescribed use did not introduce the bacteria in swabs and hence in patients where it had not already been, but probably increased the bacterial load. An increase in bacterial load increases the risk of infecting a contaminated patient and of causing a more serious disease. "But-for" the improper use of the swab, the same number of patients would be exposed to the *P. aeruginosa *bacteria, but probably fewer would have been colonised and probably fewer colonised patients would have contracted serious infections. Together with the introduction of the outbreak strain into the hospitals and the susceptibility of the patients, the reuse of the swabs can be seen as a necessary (non-redundant) condition or as a counterfactual conditional. Moreover, the improper use of the swab may fail a but-for-test (due to joint determination), but not a NESS-test. Hence, it is not clear that HCWs behavior caused the outbreak in a legal sense.

**Hospital reporting systems **for faulty medical equipment are not the same for all types of equipment. A general attitude among HCWs is that reporting is fruitless and not really necessary, especially for minor products like mouth swabs. During the investigation we learnt that several HCWs had detected discoloured or otherwise faulty swabs without reporting the event. It is worth noting that the notification of faulty swabs by an infection control nurse contributed to solving the outbreak quicker.

"But-for" the lack of reporting it is impossible to ascertain whether the outbreak would have been detected earlier as it depends on many other factors like what was reported, how it was reported in the system and what measures where taken following a report. In a probabilistic or risk assessment approach, low threshold reporting systems with appropriate follow-up routines and adequate surveillance systems, make it more likely that the contaminated swabs and the outbreak would have been detected earlier (counterfactual).

**Surveillance systems **are in place for many infectious diseases, but not for *Pseudomonas *infections [34]. Only the most prevalent or serious diseases are included into the surveillance systems after weighing factors such as costs and preventability. When the computer systems improve, more infections can be included at no or little extra cost. As concluded in the previous paragraph, had an adequate surveillance system for the *P. aeruginosa *bacterium or some of the infections it caused been in place, it is more likely that the outbreak could have been detected earlier. However, the main importance of reporting systems for faulty equipment and surveillance systems is that of preparedness. Had the swabs not been contaminated, imperfect reporting and surveillance systems do not add to the risk of causing outbreaks like unclean production of medical equipment does (counterfactual).

**The outbreak investigation **was a necessary condition to stop the outbreak. Could the number of patients affected have been smaller if the investigation had been carried out differently? During the investigation there was a tremendous pressure to find the solution quickly. A rushed investigation might have resulted in not detecting the cause or getting the results wrong, whereas a broad, systematic investigation might have taken too long causing unnecessary sufferings and deaths. As two of the authors of this article (BGI and PA) were responsible for the outbreak investigation [1,2] we are not competent to appraise the investigation.

**In conclusion**, many factors contributed to the outbreak and its eventual dimension. The main necessary condition for the outbreak was the contamination of the swabs in the production facility. The size of the outbreak measured in the number of patients affected and how long it lasted are due to several additional factors. The breaches of regulations by the producer of the swabs play an important role probably together with the reuse of the swabs in the hospital, i.e. they are conditions that are influencing the size of the outbreak. With a regulatory correct production of the swabs in the production facilities there would have been no outbreak (necessary condition). The reuse of the swabs in the hospitals and the non-optimal production probably increased the size of the outbreak (probabilistic factor). In addition, other factors that might have an influence are the lack of adequate reporting and surveillance systems.

#### Moral responsibility

Many people suffered in the outbreak. Seventy-one people with the outbreak strain died while hospitalised, and for at least 34 the investigators concluded that the *P. aeruginosa *infection probably contributed to the death. No one was found criminally liable. Several actors were in a position where they could have known and acted differently, and hence, are to be seen as morally responsible. In the discussion on causes for the outbreak two main actors emerged in the discussion on responsibility. One is the producer Snøgg AS. The other is the group of HCWs who reused the swabs and the hospital system permitting these acts or possibly even encouraging them. What is their moral responsibility?

Traditionally, medical errors have been divided into three: Unintentional error, intentional error and random mishaps. In addition, bad outcomes may happen without error [35]. For our discussion we will only focus on **unintentional error **as no one in this outbreak ever was suspected of intentionally wanting to cause harm. Unintentional error can be caused by lack of knowledge, lack of skill or non-application of relevant knowledge or skill.

**The producer **Snøgg produces a wide range of medical equipment useful for saving lives and reducing suffering. The Dent-O-Sept swab had been produced for decades and was in great demand. Their vision statement is "gjøre det enkelt å hjelpe" [Make it simple to help] . In all their appearances the producer gave no impression of intending to harm anyone, and from a virtue-ethical standpoint, the company appeared favourably (see endnote 1). When the connection between the swab and the outbreak was detected, the director of Snøgg was devastated for what his product had caused [36,37]. Some months later the company started to focus more on other factors influencing the outbreak. One of their new initiatives was to partly blame the outbreak on the introduction of the *P. aeruginosa *bacterium into the production facilities through the municipal water pipe [30]. Another was to draw the attention to the incorrect use of the swabs in hospitals [38]. The company is expected to know that they needed to have systems in place to stop bacteria in municipal tap water from reaching the end product.

The impetus not to harm patients ("Primum non nocere" – "First do no harm" ascribed to Hippocrates) and to care for the vulnerable are duties with strong deontologic bearings (see endnote 2). The duty to acquire necessary knowledge for the safe performance of health care services, as well as being precautious appear to be part of such a perspective. Hence, the actions of the producer (as well as the health care professionals) appear to breach with basic deontological bearings in health care. Moreover, if the moral norms of the producer's responsibilities are adequately regulated by law, breaking these legal regulations, such as the Act on medical devices [39], would in most cases be a breach with the moral duties of a producer.

The Dent-O-Sept mouth swab belongs to Medical device Class 1, which includes most non-invasive medical devices according to the European Council Directive 93/42/EEC [29]. The directive states that the devices must, when used, "not compromise the clinical condition or the safety of patients". "The devices and manufacturing processes must be designed in such a way as to eliminate or reduce as far as possible the risk of infection to the patient, user and third parties." Accordingly, the Council Directive represents a consequentialist approach (see endnote 3). The producer did not abide by the laws and regulations relevant to him, and thus ignoring relevant norms and relevant consequences.

The unintended error of producing contaminated swabs appears only to a small extent to result from lack of knowledge. The producer knew there had been problems in the production and had received advice on implementing QA systems which the producer had not followed in great detail. By not doing so there appears to be a non-application of relevant knowledge which would normally be characterized as negligent and culpable. However, there probably was a lack of knowledge about how bacteria can contaminate the production equipment. The Dent-O-Sept swab was the only moist item Snøgg produced. Had the microbiological quality control measures been implemented, this would probably have been revealed and harm could have been avoided. In addition, there probably also was some lack of skill in cleaning and disinfecting the wet part of production.

Hence, the outbreak was not a result of wilful or intentional error. However, the non-adherence to norms and regulations and the non-application or non-acquisition of knowledge can be conceived of as malpractice. It is not the case that science has not yet progressed enough, or that there are limitations in the predictive nature of knowledge with regards to the particular case [35].

**The health care workers **aim at saving lives and alleviate pain and suffering. Their work is legally regulated by laws and regulations, and professionally by guidelines, instructions and training. In addition, their actions are also to a large degree guided by colleagues and the culture of the workplace. One of the traditional Norwegian virtues of is that of austerity. It can partly be ascribed to the nation's economic poverty up until a few decades ago and to our Lutheran tradition of modesty ("In the sweat of thy face shalt thou eat bread", Genesis 3:19). This demand of being economical is also reflected in the Act on health personnel [40] and in instructions from the hospital management. Single use products are in conflict with being economical. In several hospitals it was accepted or even encouraged to reuse the swabs, possibly considering them to be a variant of the tooth-brush. There is also a consequentialist reasoning for this austerity: reduced cost combined with low risk.

The Norwegian and English texts on the wrap were quite different, and the Norwegian being the most ambiguous: "Antiseptisk engangspensel for munnhygiene" which literarily translates to "Antiseptic one-time-swab for mouth hygiene"; whereas the English text read: "Premoistened foam swab for mouth hygiene". Although "engangs" usually is translated to "single use" some can also understand it to be "single period-use" just like a single use syringe can be used for multiple injections in the same patient within a short time frame. To avoid possible misinterpretations a resent amendment (05.09.2007) to the European Council Directive on Medical devices has defined a "single use device" as "a device intended to be used once only for a single patient". The claim of antiseptic properties of the swab (which was never documented by the producers [17]) may have led some health care workers to underestimate the risk this practise posed. Placing a swab coated with an oral cavity bacterial flora in a glass of water with saliva and mucus as nutrition, may lead to extensive bacterial growth up to a concentration which makes it potentially harmful. And no one could presume that the swabs contained *P. aeruginosa*. In addition, there is an active debate within the medical community in Europe whether it can be safe to reuse reprocessed single use medical equipment.

There were no guidelines against this practice and no superiors contradicted it. From a virtue-ethical perspective the act was ambiguous; it was austere, but against professional standards (of following written instructions) and the duty to care for the patient. The main reason for the medical error of reusing the swab was non-application or misunderstanding of relevant information.

According to Norwegian law, hospital management shall provide for making an infection control programme, producing guidelines to prevent hospital acquired infections, having a system for surveillance of infectious diseases and for procurement and control of medical equipment. As there were deficiencies in many of these fields in many hospitals, the hospital management consequently appears to be morally negligent and legally responsible according to the NESS-test. When human error repetitively occurs within a system it is of interest to discuss whether to have a person approach or a system approach. If preventing future errors is the aim, a system approach appears to be more rewarding [41]. However, even though responsibility of the management does not free the individual employees from responsibility, it would be fruitless to try to identify individual health care workers reusing the swab and place them under moral and legal scrutiny.

After the Dent-O-Sept swab was withdrawn from the market, other similar products have taken its place. Despite all the media attention from this outbreak, we have received anecdotal information that health care workers still reuse single use mouth swabs.

#### Legal consequences

At least 231 patients contracted the bacterium and for at least 34 patients the investigators concluded that the *P. aeruginosa *infection probably contributed to the patient's death. There was much anxiety and guilt feeling among patients and relatives. Many had cared for their terminally ill relatives and used the Dent-O-Sept swab. Some called the Norwegian Institute of Public Health and asked for example: "Did I kill my mother by using the swab?"

No one was made criminally liable after this outbreak. The police started an investigation of the producer but decided not to press charges. The Norwegian Directorate for Health and Social Affairs appealed the decision several months after the time limit for appeal had expired; hence the Attorney-General could not reopen the case [42,43].

Norsk pasientskadeerstatning (NPE, the Norwegian System of Compensation to Patients) grants monetary compensation mainly for economical loss and to some degree for permanent disablement due to injury inflicted as a result of treatment in public health services in Norway [44,45]. Few patients could document economical loss because they were, among other things, elderly, disabled, not working, had severe chronic diseases or were already severely injured e.g. after serious car accidents.

By 18 February 2004, NPE had received a total of 287 claims. Of 256 claims processed 48 were accepted for compensation and 2.3 million NOK (≈ 290 000 EUR) had been awarded [46]. By June 2007 the total number of processed claims was 291 of 292. NPE sent a claim for re-compensation to the producer. In an out-of court settlement dated 11 October 2005 the producer agreed to pay NPE 1.2 million NOK (≈ 150 000 EUR) as a full and final sum of any regress demand in connection with the mouth swabs and without accepting responsibility for the outbreak (Deputy Director General R. G. Jørstad, NPE, personal communication).

In addition to the claim from NPE other civil claims were made against the producer. One large hospital reached a court settlement on 19 June 2006 with the producer and received compensation amounting to 3.3 million NOK (≈ 410 000 EUR) for additional costs incurred for prolonged hospitalisations of patients and for preventing further spread of the bacterium [47]. There may be other settlements that not have been made public. Hence the total known compensations paid by the producer amounts to 4.5 million NOK (≈ 560 000 EUR).

In jurisprudence responsibility is related to causality. To be negligent in most instances requires to have *caused *the harm. In this article we have argued that the contamination of the Dent-O-Sept swab was a necessary (non-redundant) condition for colonising and infecting the individual patients and by this "caused" the outbreak. Furthermore, there is good reason to believe that if systematic microbiological sampling of the product as part of a QA system had been in place, microbial contamination of the product would have been detected. That is, according to counterfactual probabilistic reasoning, the neglected QA system "caused" the outbreak. This claim uses both deterministic arguments (swab causes outbreak) and probabilistic reasoning (the probability that a microbiological QA system will detect the contamination). In addition many other factors contributed to the number of patients being affected (the extent of the outbreak), like the susceptibility of each individual patient being exposed, the reuse of the swab by HCWs and the hospital attitude for accepting reuse of swabs, to name a few. Several of these factors can be interpreted as INUS conditions and also play a role in applying Hill's nine viewpoints in claiming a causal association between the swab and *Pseudomonas *infection (table 1). The case also illustrates the weaknesses of the "but-for" test (with regards to assessment of cause-in-fact) as there were many concurrent factors that cannot be differentiated as necessary for the event, they were only necessary elements (NESS-test).

After establishing a cause-in-fact relationship, the proximity or adequacy of causes needs to be discussed. Whereas the contamination of the swabs during production appears to be an adequate cause, the possibility of the origin of the first bacterium through the municipal water supply is not because it is neither illegal nor verified. In addition, it precedes a "*novus actus*" which is the breech of regulations in the production. It can be argued that the reuse of the swabs in the hospitals is also proximate, at least for some of the patients becoming infected during the outbreak. Whether the individual HCW or the hospital system is responsible is open for debate [41].

Why was no one criminally charged in this outbreak? We have argued for a causal association between the contaminated swabs and *Pseudomonas *infection and death, and a breech of regulations during production has been established. However, the error was unintentional and due to a non-application of relevant knowledge and skill; a knowledge that isn't intuitively evident to everyone. The police decided not to press charges. In a press release, the police pointed at other circumstances, arguing that the health authorities had not audited the producer prior to the outbreak, and that there were irregularities in the use of the swabs in the hospitals and in the reporting of faulty medical devices. In addition, the fact that the Directorate for Health and Social Affairs did not appeal the decision until too late blocked the possibility for a reinvestigation of the case due to the procedures described in the Criminal Procedure Act.

## Conclusion

The major necessary condition causing the outbreak was the contamination of the swabs in the production facility. Without this contamination, the Dent-O-Sept outbreak would not have happened. Hence, there exists a cause-in-fact according to the but-for-test. Many other factors contributed to the outbreak and the size of it, the reuse of the single use swabs being the most important. The unintended error – by the producer of the swabs and to a minor extent by the hospital practice – was mainly due to non-application of relevant knowledge and skills, and breaches with moral duties as professionals, constituting moral negligence.

In epidemiology, other sciences, philosophy and jurisprudence there are plenty of methods and theories to explain causality and responsibility in complex situations like outbreak investigations. Applying different theories from different disciplines on the various necessary and sufficient conditions and the roles and responsibilities of the participants, is useful and important to elucidate the complex from most angles. From an outbreak investigator's viewpoint no theory is the only correct one. Using Mackie's concept of INUS conditions and Hill's nine viewpoints of claiming a causal association, applying deterministic as well as probabilistic ways of reasoning, all shed light on the issues of causality in this outbreak. Medical practice and jurisprudence is closely connected in real life as professional negligence can have legal consequences. Cases in epidemiology, such as outbreak investigation, highlight the tension both in science and jurisprudence between general causality and the causality of specific events. Moreover, applying legal theories of causation (counterfactual reasoning and the "but-for" test or the NESS test) proved important perspectives on the Dent-O-Sept outbreak.

As shown for the outbreak of *P. aeruginosa *infection the issue of causality also serves as a starting point for the debate on legal responsibility. Due to criminal procedure laws and other factors outside the discourse of causality, no one was criminally charged for the outbreak.

## Abbreviations

CE: Communauté Européenne; DNA: Deoxyribonucleic acid; EU: European Union; EUR: Euro (currency); HCW: Health care worker; ICU: Intensive care unit; INUS: Insufficient but Non-redundant parts of an Unnecessary but Sufficient condition; NOK: Norwegian kroner (currency); NPE: Norsk pasientskadeerstatning, (The Norwegian System of Compensation to Patients); QA: Quality assurance.

## Competing interests

Two of the authors (BGI and PA) were responsible for the outbreak investigation at the Norwegian Institute of Public Health. The authors declare that they have no competing interests.

## Endnotes

### Endnote 1

Virtue ethics is a branch of moral philosophy that emphasizes character as the key element of ethical thinking, rather than rules or consequences.

### Endnote 2

Deontological ethics, deontology or duty-based-ethics is an approach to ethics that focuses on the rightness or wrongness of actions themselves, as opposed to the rightness or wrongness of the consequences of those actions. The term deontology stems from Greek: deon (δέον) which means obligation or duty.

### Endnote 3

Consequentialism refers to those moral theories which hold that the basis for any valid moral judgment about an action is the consequences of the particular action. Accordingly a morally right action is an action that produces good consequences.

## Authors' contributions

BGI headed the outbreak investigation and the conception, drafting and revision of the manuscript. BH has contributed with regards to the causality theories and on the relationship between scientific and moral/legal causation, as well as revising the manuscript. PA was over all in charge of the outbreak investigation and participated in the revision of the manuscript. All authors read and approved of the final manuscript.
